# Structural aspects of enzymes involved in prokaryotic Gram-positive heme biosynthesis

**DOI:** 10.1016/j.csbj.2023.07.024

**Published:** 2023-07-24

**Authors:** Nikolaus Falb, Gaurav Patil, Paul G. Furtmüller, Thomas Gabler, Stefan Hofbauer

**Affiliations:** University of Natural Resources and Life Sciences, Vienna, Department of Chemistry, Institute of Biochemistry, Muthgasse 18, A-1190 Vienna, Austria

**Keywords:** Coproporphyrin ferrochelatase, Coproheme decarboxylase, Frataxin, Coproporphyrinogen oxidase, Uroporphyrinogen decarboxylase, Structure determination, Molecular enzymology

## Abstract

The coproporphyrin dependent heme biosynthesis pathway is almost exclusively utilized by Gram-positive bacteria. This fact makes it a worthwhile topic for basic research, since a fundamental understanding of a metabolic pathway is necessary to translate the focus towards medical biotechnology, which is very relevant in this specific case, considering the need for new antibiotic targets to counteract the pathogenicity of Gram-positive superbugs. Over the years a lot of structural data on the set of enzymes acting in Gram-positive heme biosynthesis has accumulated in the Protein Database (www.pdb.org). One major challenge is to filter and analyze all available structural information in sufficient detail in order to be helpful and to draw conclusions. Here we pursued to give a holistic overview of structural information on enzymes involved in the coproporphyrin dependent heme biosynthesis pathway. There are many aspects to be extracted from experimentally determined structures regarding the reaction mechanisms, where the smallest variation of the position of an amino acid residue might be important, but also on a larger level regarding protein-protein interactions, where the focus has to be on surface characteristics and subunit (secondary) structural elements and oligomerization. This review delivers a status quo, highlights still missing information, and formulates future research endeavors in order to better understand prokaryotic heme biosynthesis.

## Graphical Abstract

**Figure:**
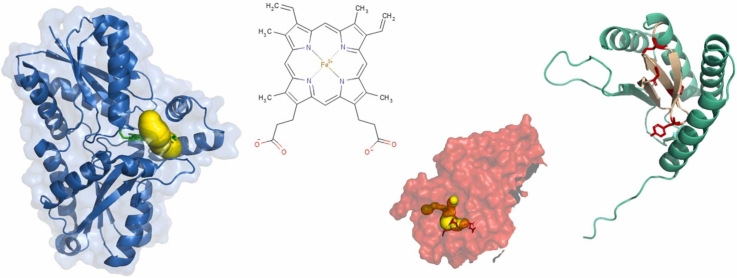

## 1 Introduction

Over the years the structural characterization of macromolecules, especially for proteins, has become an integral tool to understand and perform research on enzymatic reaction mechanisms and protein-protein interaction. This is especially true for enzymes which partake in metabolic and signaling pathways in biological systems, where structural analysis, primarily X-ray crystallography, poses an elementary tool [1], [2].

The aim of this study is to assess the availability and quality of structural data for the enzymes of the coproporphyrin-dependent (CPD) pathway of heme biosynthesis in comparison to the previously well-established protoporphyrin-dependent (PPD) pathway in eukaryotic and Gram-negative organisms. The CPD pathway was discovered in the middle of the 2010 s and is almost exclusively utilized by Gram-positive bacterial strains. It consists partially of unique enzymes and partially of structural orthologues of their PPD counterparts [3], [4], [5], [6], [7]. Why this divergence of the last three steps of heme biosynthesis has evolved in nature is unclear, one hypothesis might be the lack of cellular compartmentalization or periplasmatic space in Gram-positive bacteria [5].

**Fig. 1:**
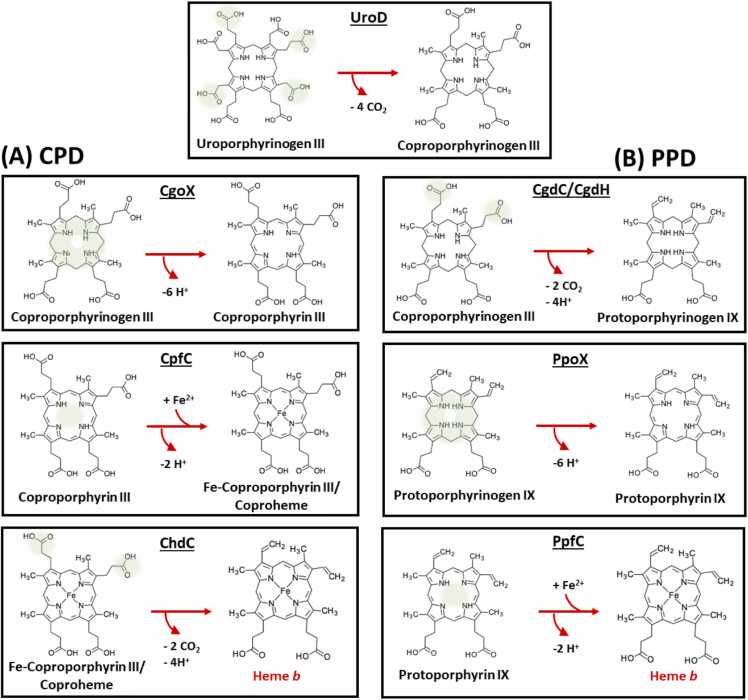
Steps of the Gram-positive CPD pathway (A) and Gram-negative/eukaryotic PPD pathway (B) from the common precursor uroporphyrinogen III and a common decarboxylation step, performed by UroD. To highlight the reaction sites on the substrate molecule, green shades mark the respective area. Chemical structures were downloaded from the KEGG compound database (https://www.genome.jp/kegg/compound/), provided by the Bioinformatics Center, Institute for Chemical Research, Kyoto University and the Human Genome Center, Institute of Medical Science, University of Tokyo. The respective identification codes are: C01051 (Uroporphyrinogen III), C03263 (Coproporphyrinogen III), C05770 (Coproporphyrin III), C21284 (Coproheme), C01079 (Protoporphyrinogen IX), C02191 (Protoporphyrin IX) and C00032 (Heme b).

The four final reactions of the PPD pathway and CPD pathway generally resemble each other, but are aligned in a different order, with the exception of a first and common step (Fig. 1): After the original precursor for heme biosynthesis 5-aminolevulinic acid (5-ALA) is synthesized by a set of different enzymes, which are universally conserved across all organisms, it then is used to synthesize uroporphyrinogen III, which leads to the before mentioned first and common step of the PPD and CPD pathway for heme biosynthesis [8], [9]. In a decarboxylation reaction, catalysed by uroporphyrinogen decarboxylase (UroD), carboxyl groups on multiple acetate side chains are removed to yield coproporphyrinogen III [10], [11], [12].

After this initial decarboxylation step the propionates 2 and 4 of the common precursor coproporphyrinogen III are decarboxylated in the PPD pathway by coproporphyrinogen decarboxylase (CgdC) or coproporphyrinogen dehydrogenase (CgdH) [4], [10], [13], [14]. A similar decarboxylation step is performed by the coproheme decarboxylase (ChdC) of the CPD pathway – a mechanistically and structurally completely different enzyme [15], [16], [17], [3]. In the PPD pathway this is followed by the oxidation and therefore aromatization of the tetrapyrrole ring of protoporphyrinogen IX by the protoporphyrinogen oxidase (PgoX), also a key enzyme in the chlorophyll biosynthesis of plants [18], [19], [20]. In the CPD pathway this is the initial step performed by the structurally highly similar coproporphyrinogen oxidase (CgoX) on coproporphyrinogen III [7], [21], [22].

The final step of the PPD pathway consists of the metal insertion into the mature porphyrin macrocycle by the protoporphyrin ferrochelatase (PpfC) [23], [24], [25], [26]. Whereas in the CPD pathway the iron insertion reaction, catalysed by the coproporphyrin ferrochelatase (CpfC), is the penultimate step [27], [28], [29].

A common shared feature of the CPD and PPD pathway is a lack of conservation in the operon organization of the pathway enzymes. The related genes can share operons or cluster through the genome. In Gram-positive genomes those operons usually consist of a few enzymes from the CPD pathway, with the others singled out at different genome sites. The number of gene ensembles in the operon depends on the organism. The genes for 5-ALA and uroporphyrinogen III synthesis form those types of non-conserved operons - the genomic organization of prokaryotic heme biosynthesis clearly lacks a universal pattern [30]. However Gram-positive organisms with a universal operon for heme biosynthesis on one genomic locus do exist, with the leading example of *Cutibacterium acnes*. An analysis of the *Cutibacterium acnes* HL096PA1 genome (source: *National Center for Biotechnology Information* NCBI) show *gttR* and *gsaM* (involved in 5-ALA synthesis), *pbgS*, *hmbS* and *uroS* (involved in uroporphyrinogen III synthesis) and finally *uroD, cgoX*, *cpfC* and *chdC* (CPD pathway) all reside next to each other between 371580 and 381804 bp. *C. acnes* also holds a special place since its ChdC and CpfC enzymes form a fusion protein, linked by a presumably flexible peptide chain [6].

In addition to the enzymes responsible for the synthesis of heme *b*, support systems for shuttling need to exist. A specific example would be proteinogenic iron transportation to CpfC to counteract the toxicity of free iron ions in the cell. Every pathway, which utilizes iron needs a suitable iron transporting protein to deliver the ions to the desired target [31], [32]. For eukaryotic organisms the iron transporting protein frataxin has been established as a transporter for Fe^2+^ and Fe^3+^ through the cell. Mutations in the sequence and the corresponding loss of function of human frataxin are connected to clinical diseases like Friedreich’s ataxia [33], [34], [35]. Meanwhile in Gram-negative bacteria, CyaY, the corresponding protein orthologue to eukaryotic frataxin has been identified for its ability to bind iron and its structural similarity to eukaryotic frataxin and its ability to supply Fe-S cluster proteins with the necessary iron [36], [37], [38]. The ability to bind and transfer iron to target recipients (like CpfC) has been shown for a homologous protein in Gram-positive prokaryotic cells, also annotated as frataxin (or protein with an YdhG domain) in *Bacillus subtilis*. Despite differences in sequence, a structural and functional conservation to the remaining frataxin family was identified [39], [40], [41]. It should be highlighted that Frataxin seems to fulfill multiple iron transport purposes, given biochemical data. This is also supported by the fact, that it is not necessarily in genomic proximity of any operons or loci containing genes for heme biosynthesis, for example in Firmicute model organism *B. subtilis* (at 621847 bp in *B. subtilis* sp. 168) and Actinobacterium *Corynebacterium diphtheriae* (at 92618 bp in *C. diphtheriae* strain ATCC 700971 NCTC 13129 Biotype gravis) (source: *National Center for Biotechnology Information* NCBI). Additionally a homologous gene for frataxin is not present in every organism, which suggests a non - essential role of frataxin in iron transfer processes [41].

The metal binding site and the amount of these binding sites has been discussed in multiple studies for CyaY and eukaryotic frataxin, using structural determination and molecular dynamics simulations [42], [43], [44], [45], [46]. The lack of studies concerning Gram-positive frataxin and if conclusions can be drawn from CyaY and eukaryotic frataxin will be discussed in the respective chapter.

**Fig. 2:**
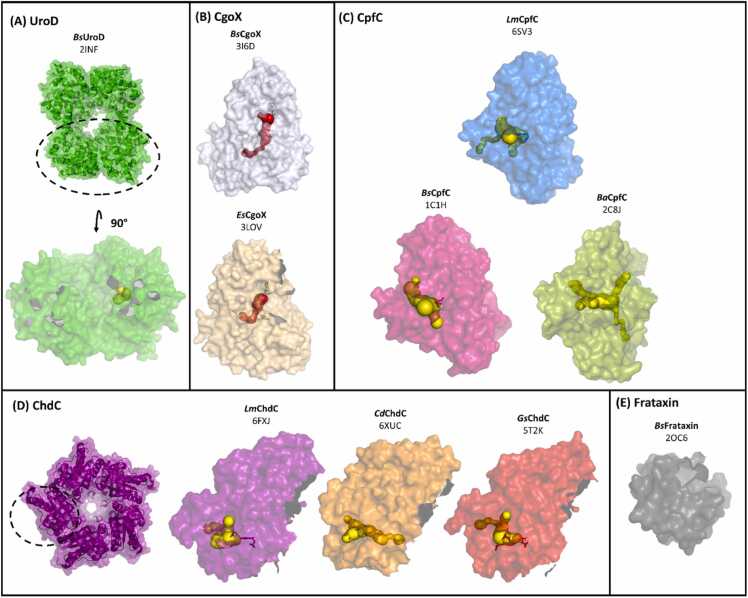
Available crystal structures of the CPD pathway in the PDB, found by keyword search. Protein structures shown are (A) UroD, (B) CgoX, (D) CpfC, (D) ChdC and (E) frataxin orthologues, which are possible iron transporters for the pathway. The figure depicts a surface overview and for (A) UroD and (D) ChdC an orientation view of the total structure/oligomer. The initial two letters of the structure titles in bold correspond to the organism of origin and are explained in the text and the glossary. PDB-IDs are depicted below. The cofactors if present are presented as sticks. Additionally, a CAVER calculation (Minimum probe radius = 0.9, Shell depth = 4, Shell radius = 3, Clustering threshold = 3.5) was performed originating from the presumed active site/cofactor binding site to further highlight its location on the protein monomer. CAVER calculations from porphyrin binding sites are depicted in yellow, for CgoX the calculations originated from the bound FAD cofactor in the structure and is depicted in red.

To summarize the known protein structures of the CPD pathway a keyword search of the Protein database (PDB) has been performed. The results are summarized in Table 1. In Table S1 the same information has been expanded by the inclusion of the provided structures of the corresponding/orthologous enzymes of the PPD pathway. This review focuses on structural and functional aspects of enzymes involved in the CPD pathway. An overview of a selection of available structures is presented in Fig. 2. Here, oligomerization status and form of the respective subunits are illustrated in surface mode. CAVER calculations based on the structures and originating from the cofactor binding sites show their conserved location on the protein body regardless of species. Further future challenges in the structural deconvolution of CPD heme biosynthesis next to unraveling enzymatic mechanisms are described as characterizing (i) the important elements of orthologous Gram-positive frataxin proteins in terms of structure and function and (ii) characterizing the interactions of proteins within the pathway by identifying the interacting structural features.

**Table 1:** Known protein structures of the CPD pathway extracted from the protein data base (www.pdb.org). Enzyme nomenclature relates to Daily et al. (2017) [47]. Table includes information about the organism (name, phylum), the crystallized protein (mutation, length) and the structure (PDB-ID, deposition date, R-free, resolution, ligands). Information was updated on 08/05/2023.

UroD/HemE (Uroporphyrinogen III decarboxylase)									
Organism	Phylum	Mutation	Length	PDB	Date	R-free	Resolution [A°]	Ligand	Notes
*Bacillus subtilis*	Firmicutes	wt	359	2INF	2006–10–06	0.251	2.3	apo	

CgoX/HemY (Coproporphyrinogen III oxidase)									
Organism	Phylum	Mutation	Length	PDB	Date	R-free	Resolution [A°]	Ligand	Notes
*Bacillus subtilis*	Firmicutes	wt	470	3I6D	2009–07–06	0.293	2.9	FAD	annoted as PPO
*Exiguobacterium sibiricum* 255–15	Firmicutes	wt	475	3LOV	2010–02–04	0.241	2.06	FAD	annoted as PPO

CpfC/HemH (Coproporphyrin ferrochelatase)									
Organism	Phylum	Mutation	Length	PDB	Date	R-free	Resolution [A°]	Ligand	Notes
*Bacillus subtilis*	Firmicutes	wt	310	1AK1	1997–05–28	0.243	1.9	apo	
*Bacillus subtilis*	Firmicutes	wt	306	1C9E	1999–08–02	0.255	2.3	Cu^2+^ N-Methylmesoporphyrin complex	
*Bacillus subtilis*	Firmicutes	wt	309	1DOZ	1999–12–22	0.216	1.8	apo	
*Bacillus subtilis*	Firmicutes	wt	310	1C1H	2000–03–17	0.231	1.9	N-Methylmesoporphyrin	
*Bacillus subtilis*	Firmicutes	wt	310	1N0I	2002–10–14	0.273	2	Cd^2+^	
*Bacillus subtilis*	Firmicutes	wt	310	1LD3	2003–05–20	0.274	2.6	Zn^2+^	
*Bacillus subtilis*	Firmicutes	Y13F	309	2AC2	2005–07–18	0.257	2.5	Zn^2+^	
*Bacillus subtilis*	Firmicutes	H183C	309	2AC4	2005–07–18	0.276	2.1	apo	
*Bacillus anthracis, str. Ames*	Firmicutes	wt	311	2C8J	2005–12–05	0.281	2.1	apo	
*Bacillus subtilis*	Firmicutes	wt	310	2H1V	2006–05–17	0.173	1.2	apo	
*Bacillus subtilis*	Firmicutes	H183A	310	2H1W	2006–05–17	0.259	2.6	apo	
*Bacillus subtilis*	Firmicutes	wt	310	2HK6	2006–07–03	0.22	1.71	Fe^2+^	
*Bacillus subtilis*	Firmicutes	wt	309	2Q2N	2007–05–29	0.25	1.8	Deuteroporphyrin IX 2,4-disulfonic acid dihydrochloride	
*Bacillus subtilis*	Firmicutes	H183C	309	2Q2O	2007–05–29	0.219	2.1	Deuteroporphyrin IX 2,4-disulfonic acid dihydrochloride	
*Bacillus subtilis*	Firmicutes	H183A	309	2Q3J	2007–05–30	0.228	2.39	N-Methylmesoporphyrin	
*Bacillus subtilis*	Firmicutes	Y13M	310	3GOQ	2009–03–19	0.232	1.6	apo	
*Bacillus subtilis*	Firmicutes	wt	309	3M4Z	2010–03–12	0.199	1.94	Co^2+^	
*Listeria monocytogenes*	Firmicutes	wt	312	6RWV	2019–06–06	0.201	1.64	apo	
*Listeria monocytogenes*	Firmicutes	wt	311	6SV3	2019–09–17	0.201	1.64	Coproheme	
*Listeria monocytogenes*	Firmicutes	R45L	311	8AW7	2022–08–29	0.213	2.64	Coproporphyrin III	
*Listeria monocytogenes*	Firmicutes	wt	311	8AT8	2022–12–28	0.182	1.51	Coproporphyrin III	
*Listeria monocytogenes*	Firmicutes	wt	311	8BBV	2022–10–14	0.242	2.19	Coproporphyrin III/2 min Fe^2+^ soak	
*Listeria monocytogenes*	Firmicutes	wt	311	8OMM	2023–03–31	0.233	2.15	Coproporphyrin III/3 min Fe^2+^ soak	
*Listeria monocytogenes*	Firmicutes	wt	311	8OFL	2023–04–05	0.229	2.1	Coproporphyrin III/4 min Fe^2+^ soak	

ChdC/HemQ (Coproheme decarboxylase)									
Organism	Phylum	Mutation	Length	PDB	Date	R-free	Resolution [A°]	Ligand	Notes
Thermus thermophilus HB8*	Deinococcota	wt	249	1VDH	2004–03–22	0.218	2	apo	*Gram - negative
*Geobacillus stearothermophilus*	Firmicutes	wt	248	1T0T	2004–04–12	0.194	1.75	apo	
*Listeria monocytogenes*	Firmicutes	wt	253	4WWS	2014–11–12	0.229	2	apo	
*Listeria monocytogenes*	Firmicutes	wt	250	5LOQ	2016–08–09	0.215	1.69	Coproheme	
*Geobacillus stearothermophilus*	Firmicutes	wt	248	5T2K	2016–08–23	0.176	1.8	Mn-Coproporphoryn III	
*Listeria monocytogenes*	Firmicutes	wt	250	6FXQ	2018–03–09	0.214	1.69	Coproheme, monovinyl monopropionate deuteroheme	
*Listeria monocytogenes*	Firmicutes	wt	250	6FXJ	2018–03–09	0.214	1.79	Coproheme	
*Corynebacterium dipththeriae*	Actinobacter	wt	237	6XUB	2020–01–17	0.227	1.78	monovinyl monopropionate deuteroheme	
*Corynebacterium dipththeriae*	Actinobacter	wt	237	6XUC	2020–01–17	0.223	1.87	Coproheme	
*Geobacillus stearothermophilus*	Firmicutes	wt	248	6VSA	2020–02–10		2.32	apo	Cryo EM
*Geobacillus stearothermophilus*	Firmicutes	wt	248	6VSC	2020–02–11		2.6	apo	Cryo EM
*Corynebacterium dipththeriae*	Actinobacter	Y135A	237	7Q4G	2021–10–30	0.219	1.82	Coproheme	
*Corynebacterium dipththeriae*	Actinobacter	W183Y	237	7Q4F	2021–10–30	0.182	2.15	Coproheme	

Frataxin									
Organism	Phylum	Mutation	Length	PDB	Date	R-free	Resolution [A°]	Ligand	Notes
*Bacillus subtilis*	Firmicutes	wt	124	2OC6	2006–12–20	0.217	1.75	apo	Hypothetical protein (NP_388456.1)
*Lactobacillus casei*	Firmicutes	wt	123	2I8D	2006–09–01	0.197	1.69	apo	Hypothetical protein (ZP_00384875.1)
*Alkalihalobacillus halodurans*	Firmicutes	wt	118	2KL4	2009–06–30			apo	NMR

Table 1 and Table S1 include information about the organism of origin, if wild-type or variants were studied, deposition date, R-free and resolution and presence of ligands. It is intended to be a starting point for open research questions concerning mechanism and structures for this set of enzymes. R-free and resolution serve for general quality assessment but not necessarily indicate the fitness of the dataset to tackle the specific question of the respective study.

## 2 The enzymes of the CPD pathway in detail

### 2.1 UroD: The common step

**Fig. 3:**
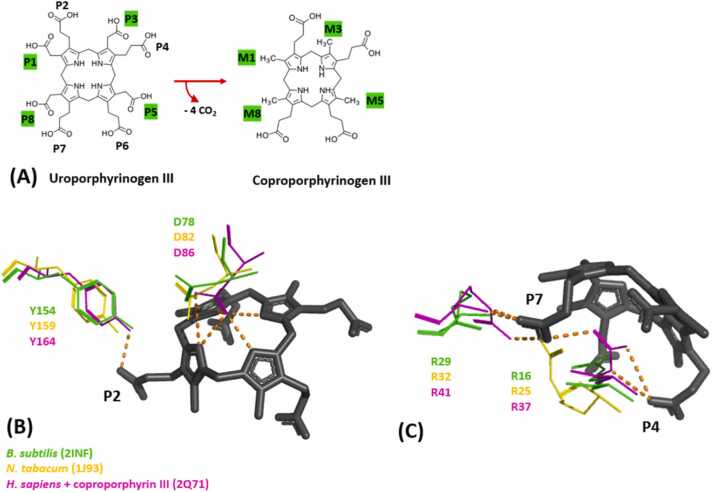
(A) General reaction catalysed by UroD, with labeled propionate (P) residues. Remaining methyl groups in the product are labeled (M), side chains changing during reaction are highlighted in green. (B & C) Cofactor binding site overlay of UroD from *B. subtilis* (2INF) (green), *N. tabacum* (1J93) (yellow) and *H. sapiens* (2Q71) (magenta). 2Q71 is a crystal structure with a coproporphyrin III molecule (black) bound, and the hydrogen bonds it forms with nearby residues are highlighted in orange. (B) shows distal site of the cofactor with tyrosine and aspartate residues visible, (C) shows the proximal site of the cofactor with two arginine residues visible.

The last common step in prokaryotic and eukaryotic heme biosynthesis is catalysed by UroD. It catalyses the decarboxylation of acetate side chains of the intermediate uroporphyrinogen III. It also holds a special place, since it is a non-oxidative decarboxylase, without the utilization of any cofactor. During reaction the 8-propionate substrate uroporphyrinogen III is degraded to a 4-propionate substrate, namely coproporphyrinogen III (Fig. 3A). The relevant propionates are left as methyl groups after decarboxylation [48], [49], [50], [51], [52], [53].

Currently 27 crystal structures are deposited in the PDB (Table S1). Several mutations and the wild-type of the human enzyme were structurally characterized. Six available structures are UroDs of Gram-negative origin. The single crystal structure of a UroD from Gram-positive heritage was described by Fan et al. in 2008 (2INF) (Table 1**, S1**). The resolution is at 2.3 Å and the R-free value is 0.251, all relevant amino acid residues discussed below are resolved, which makes it suitable for analysing structure and function and also putting it in context with known crystal structures from different organisms [49].

The enzyme forms homodimers of approximately 80 kDa in total, with a conserved active site cleft surrounded by a flexible region (Fig. 2A) [49], [52]. This structural arrangement as a dimer and with a flexible loop partially covering the active site has been hypothesized to be a feature to keep the reaction intermediate inside, and also to require substrate reorientation for the sequential decarboxylation of the acetate groups [52], [50]. The current state of knowledge suggests a clockwise rotation and order of decarboxylation at physiological concentrations of uroporphyrinogen III [50].

The residues which are assumed to be catalytically important, are mostly conserved in all organisms and consist of an aspartate residue, which seems to be important for binding the pyrrole ring of the substrate (D78). Polar residues (R29, R33, Y154, H322) seem to be of importance for substrate binding and recognition and side chain interactions. H322 is proposed to be decisive for the orientation of the partially decarboxylated product. Besides the histidine residue, the aspartate residue has been discussed being a key residue in catalysis and being involved in the protonation of methylene intermediate side chains, as the final step of forming methyl groups. The tyrosine residue is also assumed to be involved directly or indirectly in protonation and weakening of the carboxyl group linkage [49], [52], [53]. It must be noted that the information on the single residues is only based on the crystal structures of UroD from other organisms, which also partially have a coproporphyrin ligand bound in the active site and molecular modeling approaches were based on them [51], [52]. Comparable data does not exist for any prokaryotic version of the enzyme (Table S1) and a crystal structure with uroporphyrinogen III bound does not exist yet. The active site is depicted in Fig. 3B & C with the most relevant residues shown as lines. As mentioned, the bound cofactor is not uroporphyrinogen III, but rather a coproporphyrin III molecule and all interactions are subsequently discussed in respect to this fact. Overall, the porphyrin is subjected to bended conformation with the aspartate (D78/82/86) residue coordinating the center and the tyrosine residue (Y154/159/164) coordinating propionate 2 on the distal site. (Fig. 3B) All residues align positionally in the crystal structure regardless of the source organism, however the presence of the cofactor in 2Q71 induces conformational differences, given the presence of polar interactions. On the distal site (Fig. 3C) one arginine residue interacts with propionate 4 (R16/25/36) and the other interacts with propionate 7 (R29/32/41) The histidine residue mentioned above is not found near coproporphyrin III and is not included in the Figure. Since literature suggests the sequences and structures of UroDs from the PPD and the CPD pathways align, therefore it seems to follow a similar reaction mechanism, regardless of the organism.

### 2.2 CgoX: Tetrapyrrole oxidation in the CPD pathway

**Fig. 4:**
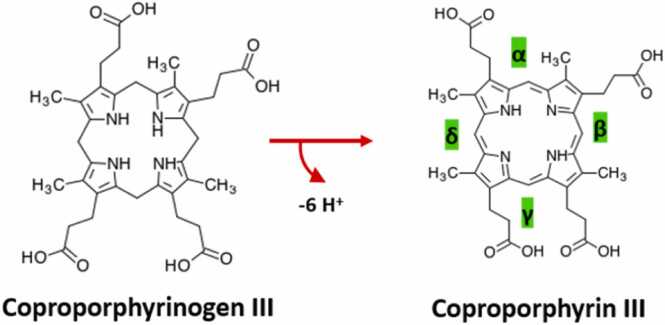
Reaction catalysed by CgoX. The formed methyl groups which connect the macrocyclic tetrapyrrole ring are labeled by Greek letters (α,β,γ,δ) and marked in green to highlight them as reaction sites.

CgoX catalyses the oxidation of the pyrrole rings and the bonds connecting them, making it an enzymatic reaction affecting the full porphyrin. (Fig. 4) [7], [21]. A graphical analysis of the resolved crystal structures was left out, due to the lack of a porphyrin bound structure of CgoX and therefore no definite knowledge about the important residues for coproporphyrinogen III binding and the subsequent reaction.

Currently two crystal structures of CgoX are deposited in the PDB (Table 1*,* Fig. 1): Both are annotated as PgoX, which is due to their deposition date (2009 and 2010), back then the CPD pathway was not described yet. The crystal structure of CgoX from *B. subtilis* (3I6D) (*Bs*CgoX) was released by Qin et al. in 2010. Alongside the structure of CgoX from *Exiguobacterium sibiricum 255–15* (3LOV) (*Es*CgoX) the monomeric form of CgoX is confirmed. This monomeric state of the soluble protein is discussed as a unique feature of *Bs*CgoX, compared to e.g.: human CgoX, which forms membrane bound dimers [7]. Both structures have a flavin adenine dinucleotide (FAD) cofactor and 3I6D has an additional acifluorfen inhibitor bound. As it is the case for UroD, most structures deposited in the PDB are homologs originating from eukaryotes or Gram-negative bacterial strains. 13 from 15 structures for the tetrapyrrole oxidizing enzyme in heme biosynthesis are the orthologous protein PgoX from the PPD pathway (Table S1).

Little is known about the exact reaction mechanism, recently a non-conserved aspartate residue in *Bs*CgoX has been shown to be important to maintain an intact H-bonding network in close proximity to the FAD cofactor [54]. The key catalytic residues of CgoX have not been discussed yet – however its ability to utilize coproporphyrinogen III as a substrate has been postulated. Interestingly different bacterial strains that possess CgoX are also able to utilize protoporphyrinogen IX as a substrate in varying degrees [7], [3]. However even before the discovery of the CPD pathway, a higher affinity of coproporphyrinogen III to CgoX was discussed based on the crystal structure of *Bs*CgoX [7]. Since there is no direct homolog in the PPD pathway, CgoX is of particular interest for potential inhibitor studies: Besides acifluorfen, which inhibits PgoX [55], [56] and inefficiently inhibits *Bs*CgoX [7] and CgoX from *Staphylococcus aureus* (*Sa*CgoX) a variety of other herbicides have shown inhibitory capacity for the enzyme. [3] Furthermore it has been of interest as a target for vaccination against Gram-positive pathogens and other therapies [57], [58]. However, a better understanding of the reaction mechanism of CgoX through structural analysis plays an important role in the development of new anti-bacterial compounds [59].

### 2.3 CpfC: Metal insertion

Most of the CpfC structures available are from *B*. *subtilis* (*Bs*CpfC) and are annotated as HemH, since their depositions backdate (1997 – 2010) to a time when the CPD pathway was not yet discovered. A wide variety of divalent metals were used to find metal binding sites as well as non-physiological porphyrins in the crystallization attempts, which consequently partially bound incorrectly in the pocket of CpfC, but still provided important information [60], [61], [62], [63], [64], [65]. CpfC structures in context of the true physiological role are available from *L*. *monocytogenes*, providing all three possible physiological states: apo, coproporphyrin III and iron coproporphyrin III bound (6RWV; 8AT8; 6SV3) (Table 1) [27], [66], [67].

CpfC is a monomer most likely located in the bacterial cytosol, compared to e.g.: human PpfC which is a homodimer containing a [2Fe-2S] cluster in each subunit coordinated by four cysteine residues and is membrane-associated in the mitochondria. [68], [69], [70], [71] 2Fe-2S] clusters seem to be present also in CpfC representatives of the actinobacterial clade for instance *C*. *diphtheriae*, but are not conserved within the clade and not present in firmicute CpfCs [72]. The role of the cluster in bacteria is under debate, since the metalation of coproporphyrin III in Firmicutes is performed without it at no catalytic cost [28], [47], [73].

The porphyrin substrate is coordinated by a variety of amino acid residues in the active site by an extensive hydrogen bonding network interacting with the propionate sidechains and is clearly distorted [64], [67], [74]. A distortion of the substrate was postulated and shown to be necessary by QM/MM calculations to be energetically favorable, however they were not performed with the physiological substrate coproporphyrin III, but with different porphyrin analogues [26], [75], [76].

**Fig. 5:**
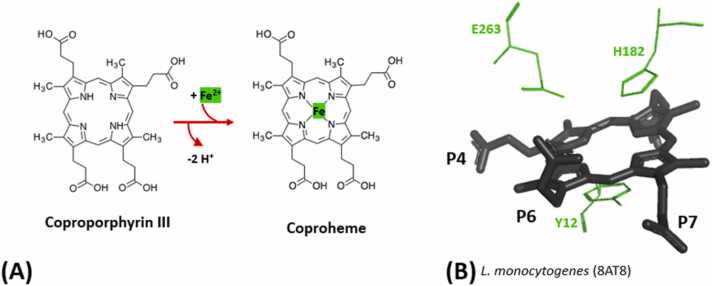
(A) Reaction catalysed by CpfC. Inserted Fe^2+^ is highlighted in green. (B) Active site of CpfC from *Listeria monocytogenes* (8AT8). The cofactor coproporphyrin III is shown in black with visible propionates labeled (P) for orientation. Catalytically important residues are shown as green lines. On the proximal side a tyrosine residue is located and on the distal side a glutamate and a histidine residue are visible.

For the iron insertion process two protons have to be abstracted from the pyrrole nitrogen atoms and one electron has to be transferred to oxidize Fe(II) to Fe(III) during the ligation (Fig. 5A). The three prominent amino acid residues are a glutamic acid (E263) and a histidine (H182) on the distal side and a tyrosine (Y12) located on the proximal side (Fig. 5B). Those residues are believed to be important or essential for these tasks. The residues at the distal site are highly conserved throughout all ferrochelatases and it was reported that the histidine variant showed a highly reduced catalytic capability [62], [61], [77], [78], [79]. It is important to mention that these observations were reported using protoporphyrin IX, which has two vinyl instead of two propionate groups. Whether this holds true for the physiological substrate coproporphyrin III remains to be shown. The proximal residue is conserved within the phylogenetic clades. A tyrosine can be found in Firmicutes and a phenylalanine in actinobacterial representatives, whereas in human PpfC a methionine is present [27], [72], [47].

The path and binding site of the iron to the active site prior to the ligation into coproporphyrin III is of utter interest since it can indicate amino acids involved in the ligation process. In an earlier structure of apo *B*. *subtilis* the iron (2HK6) was located between the prominent distal glutamic acid and histidine, but no bound porphyrin was present [61]. In contrast, the QM/MM calculations mentioned above with different porphyrin analogues, suggested that the proximal path is more favorable, as also some investigations of the structurally related human ferrochelatase [26], [69], [80]. There are three new structures available of *L*. *monocytogenes* with coproporphyrin III soaked with ferrous iron resolved with different iron occupancies (8BBV; 8OMM; 8OFL, Table 1). These potentially foreshadow an iron approach from the proximal side and conformational changes of the substrate coproporphyrin III during the ligation process. Yet, no proven conclusion can be drawn.

Despite new data and progress, important questions of about this enzymatic reaction remain under investigation: (i) How are the protons abstracted? (ii) From where does the iron approach and (iii) whereto is the electron transferred during the oxidation process of iron?

### 2.4 ChdC: Terminal two-propionate decarboxylation

**Fig. 6:**
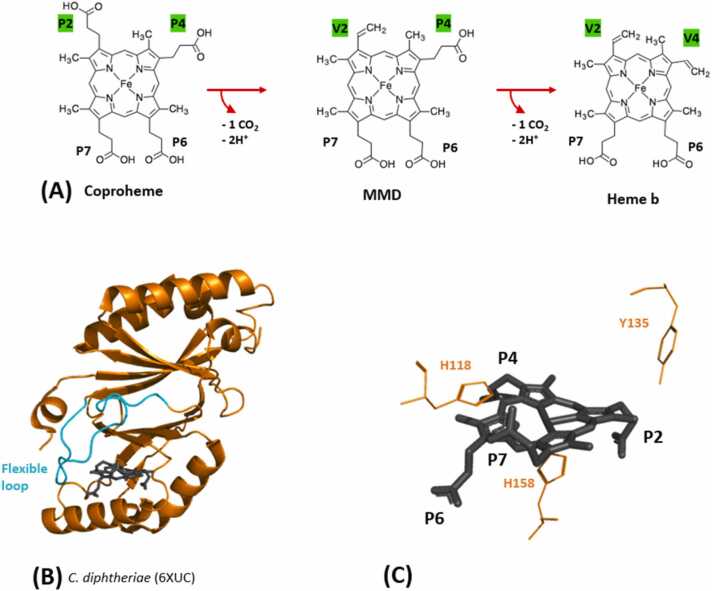
(A) Reaction catalysed by ChdC. Original propionate groups and emerging vinyl groups are labeled (P,V). Sidechains which are reaction sites are marked in green. Included in the reaction is also the three-propionate reaction intermediate MMD. Chemical structure was downloaded from the KEGG compound database (*https://www.genome.jp/kegg/compound/*), provided by the Bioinformatics Center, Institute for Chemical Research, Kyoto University and the Human Genome Center, Institute of Medical Science, University of Tokyo. The identification code is C22173. (B) Monomeric subunit of ChdC from *Corynebacterium diphtheriae* (6XUC) with black coproheme cofactor marking the active site. Flexible linker is distinctly colored in cyan (C) Cofactor in the active site with propionates labeled for orientation (P). Visible residues are a histidine and a tyrosine on the distal site and a histidine on the proximal site.

Coproheme decarboxylases (ChdCs) catalyse the final step in heme *b* biosynthesis of monoderm and some diderm bacteria. [47] In this reaction, coproheme is converted to heme *b* via monovinyl monopropionate deuteroheme (MMD) in two consecutive decarboxylation steps (Fig. 6A). The oxidative decarboxylation of coproheme is in the physiological setting most probably hydrogen peroxide mediated and requires two equivalents of the oxidant (e. g. hydrogen peroxide, in vitro also chlorite and peroxyacetic acid were used) for full conversion of one coproheme to heme *b*. [81] A tyrosine residue was identified as the catalytically relevant radical site essential for both decarboxylation reactions in ChdCs from the Firmicutes *Staphylococcus aureus* and *Listeria monocytogenes* and also from actinobacterial *Corynebacterium diphtheriae* (Y135 in Fig. 6A) [82], [83]. Additionally in Actinobacteria a histidine residue, which is not conserved in firmicute representatives, was identified to act as a distal base in ChdC from *Corynebacterium diphtheriae* (H118 in Fig. 6C) [84]. Structural biology was essential in delivering key information on several important details in understanding of the catalytic reaction mechanism of ChdCs. In combination with in-solution spectroscopic studies and computational investigations, X-ray crystallographic data revealed insight, especially into the reorganisation mode of the transiently formed three propionates intermediate MMD. When coproheme is bound the catalytic tyrosine is in close proximity to p2 of pyrrole ring A, whereas after the first decarboxylation step MMD rotates in situ and places p4 of pyrrole ring B close to the catalytic tyrosine. [82], [84], [85], [86], [87].

All ChdCs are homopentamers (135–165 kDa) and have a subunit size of 250–300 amino acids that form an N-terminal and a C-terminal ferredoxin-like fold. Only the C-terminal domain has a functional coproheme/heme *b* binding site (Fig. 6B). While most β-sheets and α-helices overlay nicely between different species, the loop region combining the N- and the C-terminal domain of coproheme decarboxylases shows some differences. This loop defines the putative substrate access channel (for coproheme or heme *b*, and hydrogen peroxide or chlorite, respectively) (Fig. 6B) [88].

ChdC structures of five organisms are deposited in the PDB at resolutions ranging from 1.69 Å to 2.6 Å (Table 1); from *Geobacillus stearothermophilus*, *Listeria monocytogenes*, *Thermus thermophilus* (1VDH), *Thermoplasma acidophilum* (3DTZ) and, *Corynebacterium diphtheriae.*

Apo structures are available from *Geobacillus stearothermophilus*, solved by X-ray crystallography (*Gs*ChdC, 1T0T) and cryo-electron microscopy (6VSC, 6VSA), and *Lm*ChdC (4WWS); holo structures from *Gs*ChdC in complex with Mn-coproheme (5T2K) and *Lm*ChdC in complex with iron coproheme (6FXJ) and the three-propionate intermediate (6FXQ). Recently the structures of coproheme decarboxylase from *Corynebacterium diphtheriae* were determined in complex with coproheme (6XUC) and in complex with monovinyl monopropionyl deuteroheme (6XUB) along with various mutants from *Cd*ChdC [16], [82], [84], [89], [90], [91]. By listing all available deposited structures, it becomes obvious that no structure of any heme *b* bound ChdC has been solved until now. It would be important to have structural information of all enzymatically relevant states (empty, substrate, intermediate and product bound) of ChdC in hand (apo-ChdC, coproheme-ChdC, MMD-ChdC, heme *b* ChdC).

## 3 Cellular iron transport by YdhG/Frataxin like proteins

**Fig. 7:**
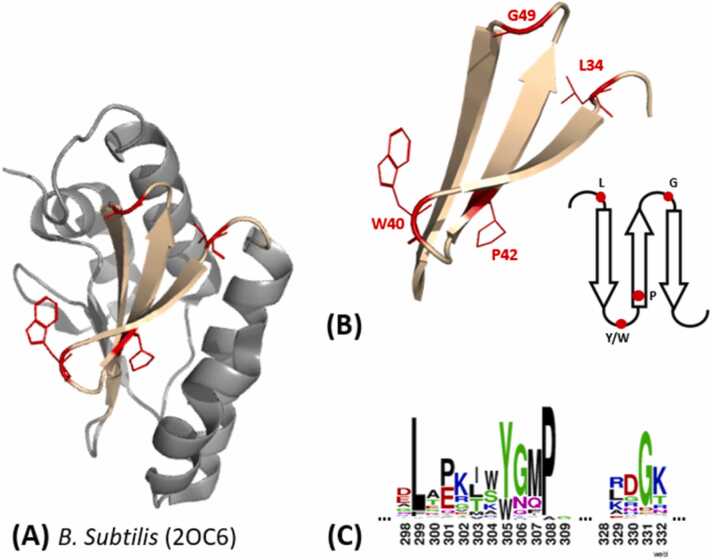
(A) Crystal structure of *Bs*Frat (2OC6), with highlighted conserved residues in red, conserved antiparallel beta sheet (wheat) and alpha helices (gray). (B) Close Up of conserved antiparallel sheet region (cartoon in wheat), with conserved residues presented as red lines. An additional scheme shows their structural positions in the sheet, according to 2OC6 and the sequence alignment in (C). (C) Results of the multiple sequence alignment of the relevant sequence range, depicted by *Berkeley WebLogo*. The conserved leucine residue is at alignment position 299, the conserved tyrosine/tryptophan residue at 305, proline at 308 and glycine at 331.

As mentioned before, the intracellular transfer of iron ions is dependent on protein transporters to protect cells from toxicity. For the CPD pathway this role might be fulfilled by frataxin, an analogous protein to eukaryotic frataxin and an orthologous protein to Gram-negative and prokaryotic CyaY [39], [40], [41]. Little is known so far about the mechanism of Gram-positive frataxin. Studies highlight its capacity to bind and transfer iron to CpfC and other proteins utilizing iron-sulfur clusters [39], [40]. However available structural data is sparse, since the stoichiometry of iron binding, the specificity for iron as a binding partner and the exact location of bound atoms on the protein has not been determined yet. Currently three crystal structures in of apo-forms are available in the PDB (2I8D, 2KL4, 2OC6), all three are of firmicute origin and one of them (2KL4) is an NMR structure. Fig. 7.

**Fig. 8:**
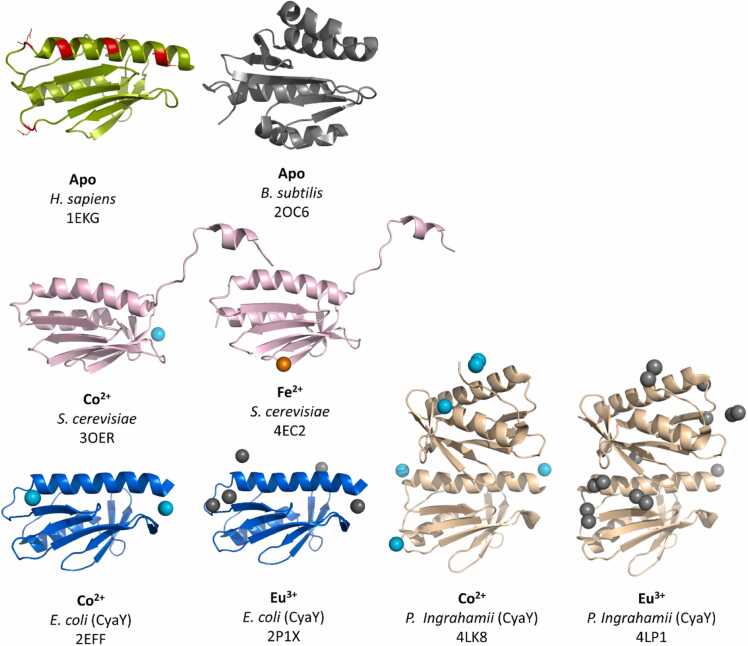
Known frataxin and CyaY crystal structures in cartoon depiction and from the same perspective to allow comparison of metal binding sites. Structures include: Apo-frataxin from *Homo sapiens* (splitpea) with possible residues involved in metal binding highlighted in red (1EKG), apo-frataxin from *Bacillus subtilis* (black) (2OC6), Co^2+^ bound frataxin from *Saccharomyces cerevisiae* (lightpink) and Co^2+^ in cyan (3OER), Fe^2+^ bound frataxin from *Saccharomyces cerevisiae* in (lightpink) and Fe^2+^ in orange (4EC2), Co^2+^ bound CyaY from *Escherichia coli* (marine) and Co^2+^ in cyan (2EFF), Eu^3+^ bound CyaY from *Escherichia coli* (marine) and Eu^3+^ in black (2P1X), Co^2+^ bound CyaY from *Psychchromonas ingrahamii* (lightorange) and Co^2+^ in cyan (4LK8), Eu^3+^ bound CyaY from *Psychchromonas ingrahamii* (lightorange) and Eu^3+^ in black (4LP1).

To pre-analyse possible conserved structural elements in Gram-positive frataxin a multiple sequence alignment of 330 sequences of possible candidates of Gram-positive origin has been performed (in *MEGA11* by *ClustalW* algorithm). A key motif in all these proteins seems to be an arrangement of three antiparallel β-strands with conserved residues interspersed (Fig. 8B & C): A leucine residue, a tyrosine or tryptophan residue, which are both polar and aromatic amino acids and a proline and a glycine residue (L34, W40, P42, G49 in *B. subtilis*). This structural arrangement is found in all three- Gram-positive frataxin structures. It must be noted that Mielcarek and co-workers identified the possible interaction site between frataxin from *B. subtilis* (*Bs*Frat) and the CpfC from *B. subtilis* (*Bs*CpfC) by hydrogen-deuterium exchange experiments and showed that the binding site is not necessarily located at the described conserved motif [40]. This highlights the need for more structural data on Gram-positive frataxin, mainly a metal-bound structure to identify the binding site is lacking. Co-crystallization experiments with CpfC can contribute to increase understanding of the role of iron transporters within the CPD pathway.

For the iron and metal binding sites, reviewing the variety of metal bound structures from eukaryotic frataxin and CyaY of different origins it might be possible to draw conclusion for Gram-positive frataxin. While sequence homology of Gram-positive frataxin to its relatives is not conserved, a certain degree of structural conservation seems to be maintained across species: When comparing the respective monomers (Fig. 8), the antiparallel sheet conformation, covered by alpha - helices seems to be maintained. This is important, giving the flexible linkers between the sheets and the surface pockets they form with the helices, seem to host metal binding sites frequently (see binding site for Co^2+^ in 3OER, 2EFF, 4LK8 and Eu^3+^ binding sites in 2P1X, 4LP1).

In general frataxin and CyaY bind different types of metal with different charges [42], [43], [44], [45], [46]. Zn^2+^ binding in human frataxin has been confirmed by NMR spectroscopy at the same binding sites as Fe^2+^. The ability of metal loaded frataxin to bind protoporphyrin IX has been shown in the same publication - a feature which highlights the ubiquitous binding modes of frataxin. Whether this is related to any physiological function is not clear. Further, seven possible residues, which change conformation during metal titration and may serve as possible binding sites, are identified by molecular dynamics simulations. Most of them possess polar side chains and are located on the N-terminal alpha helix or the flexible areas in between the sheet conformation (see red residues in Fig. 8, 1EKG). [42] The N-terminal helix and the following first strand is discussed in other publications and for other organisms as well. In a recent publication Rodrigues et al. (2022) highlights the metal binding sites on it for the *Drosophila melanogaster* frataxin variant [92]. This site for polar and anionic residue site is conserved in yeast frataxin, *E. coli* CyaY and Gram-positive *B. subtilis* frataxin. [40], [93], [94].

This suggests in consequence that metal binding of frataxin and frataxin-like proteins is solvent exposed and does not necessarily happen in a 1:1 ratio. Crystal structures from the CyaY family seem to support this conclusion, since they show several metal ions across the molecules with no uniform binding mode even between the two monomers in one unit cell. (see Fig. 8, 2EFF, 2P1X, 4LK8, 4LP1) [43], [44], [93].

Following up on the oligomeric status of frataxin, it has been shown that metal binding supports oligomerization for the apo-monomers of eukaryotic frataxin from *S. cerevisiae* (see Fig. 8, 3OER, 4EC2): Co^2+^ binds in the 3-fold axis of a formed trimer, which has been shown by SAXS (Small angle X-ray scattering) measurements, and at the binding site published in the crystal structure (3OER). Also hexameric and dimeric formations have been detected. [46] Fe^2+^ has a similar effect on the oligomerization status. In addition, SAXS measurements suggest a tetrameric form after Fe^2+^ binding [45].

Coming back to Gram-positive frataxin, it can be assumed that iron and metal binding generally happens in a similar manner as for eukaryotic frataxin and CyaY. This is mainly based on protein structure similarities. Further biochemical data suggests a 2:1 frataxin:Fe^2+^ relation, which suggests dimerization as a consequence of metal binding [41]. Therefore a metal-bound structure would not only confirm previous assumption about metal binding sites, but also its oligomerization behavior and its respective conformation.

## 4 Outlook: Enzyme - enzyme interaction as a future challenge

Crucial open questions concern (i) how the described enzymes interact with each other mechanistically, (ii) in which way they have direct influence on the metabolic flux of precursors to heme *b* and (iii) what are the tools and support systems that enable functionality in their interaction partners. The exact mode of action of an enzyme interaction strongly depends on the localization within the cell and the corresponding conditions. This also implies the lack of direct enzyme-enzyme interactions in many pathways and the inclusion of a transporter into the pathway [95], [96]. A relevant example for an interactional study including a multi-protein complex is the description of compartmentalized heme biosynthesis in eukaryotes, which utilizes a variety of proteins within and outside of the mitochondrial membrane, who have many possible interaction modes [97], [98].

This highlights that enzyme-enzyme interactions especially in a metabolic pathway are multifaceted and complex problems. In order to study such interactions many things must be considered: (i) Does one deal with a multi-enzyme complex? (ii) Do interactions happen transiently between proteins in the cytosol? (iii) Is substrate shuttling dependent on the proteins interacting or solely on a concentration gradient of the substrate [95]? To tackle those questions and problems for the CPD pathway we suggest a structure-based approach to study enzymatic interactions, with a focus on the interaction between CpfC and ChdC, the last two enzymes of the pathway.

**Fig. 9:**
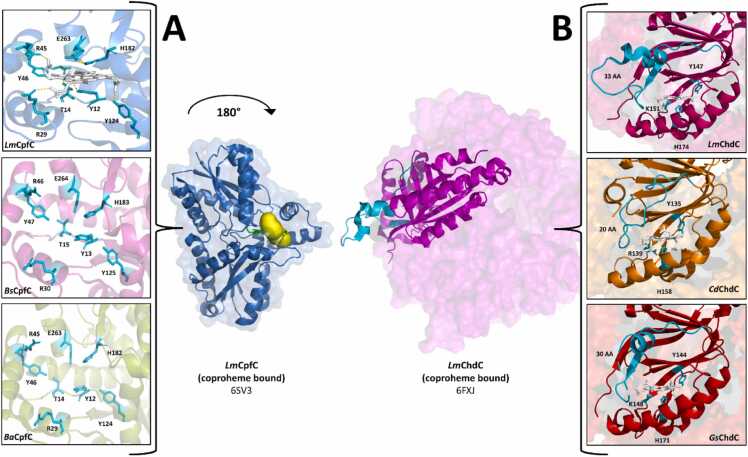
(A) Active sites of coproporphyrin ferrochelatases and overall monomeric structure, represented for *Lm*CpfC (blue). (B) Active sites and orientation of flexible loop regions for various coproheme decarboxylases and overall pentameric structure with highlighted subunit, represented for *Lm*ChdC (magenta).

The current state of knowledge is rather limited in order to be able to make elaborate guesses, based on the location of the respective active sites within the protein and their active site architectures. Coproheme shuttling from CpfC to ChdC has been confirmed by steady-state and pre-steady-state assays [5], [66]. Therefore the flexible binding loop covering the ChdC active site is very likely part of the interacting surface, when enzymes collide or bind to each other [15], [88]. However the positioning and possible motion of the loop during the interaction is not clear yet and is a key target for future structure-function studies of the CpfC-ChdC interaction. In previous works it has been shown that the loop length and orientation is highly flexible and important for substrate specificity [15], [88]. Therefore, this loop remains an interesting feature to be investigated regarding putative protein-protein interactions (Fig. 9). Further CpfC and ChdC have shown interaction with IsdG, a heme oxygenase, which seems to be relevant for regulating heme biosynthesis in *S. aureus*. [99]. Besides the biochemical characterization of this regulatory interaction, a structural investigation of a potential interaction might be of interest for gaining a deeper understanding of the CPD pathway.

The most difficult, but key task would be an experimental structure of the interacting enzymes either co-crystallized in complex or using a Cryo-EM based approach. Both methods have been used previously to study protein-protein interactions and can in combination with *in vitro* kinetic assays and *in vivo* growth and function-based assays lead to a good overview and understanding of an interaction [100], [101], [102], [103], [104], [105], [106]. Also computational methods, like molecular dynamics simulations, have been a suitable approach to describe the movement and changes of macromolecular arrangements [107], [108]. This includes elaborated docking calculations on *AlphaFold2* models, a popular machine learning algorithm, which can accurately predict structures based on protein sequences, although without co-factors and other interaction sites. Protein complex prediction itself is currently not a reliable feature of *AlphaFold2*. [109], [110], [111], [112]. Such a computational approach could be a valid alternative and add-on to study very transient interactions.

Overall, in order to understand the reactions in a metabolic pathway, enzymatic interactions are key determinants for rate, function and order and are therefore a necessity when attempting to fully understand the CPD pathway.

## Supporting

Supporting information contains Table S1, listing all structural data of enzymes involved in either the CPD or the PPD pathway, extracted from the protein data base.

## Author statement

The work was written, reviewed and edited by all authors (NF, GP, PGF, TG, SH). Conceptualization of the work was done by NF and SH. Graphical illustration by NF. Data mining by NF.

## Funding

This project was funded by the Austrian Science Funds (FWF) projects W1224, P33544, P34934, P36967.

## Declaration of Competing Interest

The authors of the submitted manuscript “Structural aspects of enzymes involved in prokaryotic Gram-positive heme biosynthesis” declare no conflict of interests.
