# Supplementary material for: Structural aspects of enzymes involved in prokaryotic Gram-positive heme biosynthesis
Source: Comput Struct Biotechnol J. 2023 Jul 24;21:3933–45. doi: 10.1016/j.csbj.2023.07.024 (PMC10427985; doi:10.1016/j.csbj.2023.07.024)
Supplement: Supplementary file 1 — Supplementary material [file mmc1.pdf]

Supporting Information

Structural aspects of enzymes involved in prokaryotic heme biosynthesis

Nikolaus Falb, Gaurav Patil, Paul G. Furtmüller, Thomas Gabler, Stefan Hofbauer*

University of Natural Resources and Life Sciences, Vienna, Department of Chemistry, Institute of Biochemistry, Muthgasse 18, A-1190 Vienna, Austria

***Correspondence: stefan.hofbauer@boku.ac.at**

Table S1. Known protein structures of the CPD pathway extracted from the protein data base (www.pdb.org). Enzyme nomenclature relates to Daily et al. (2017)[47]. Table includes information about the organism (name, phylum), the crystallized protein (mutation, length) and the structure (PDB-ID, deposition date, R-free, resolution, ligands). Information was updated on 08/05/2023.

UroD/HemE (Uroporphyrinogen III decarboxylase)

Organism	Phylum	Taxonomy	Mutation?	Length	PDB code	R-free	Resolution [A°]	Ligand	Notes
Bacillus subtilis	Firmicutes	**prok g+**	wt	359	2INF	0.251	2.3	apo	
Shigella flexineri	Pseudomonadota	**prok g-**	wt	354	3CYV	0.282	2.8	apo	
Nicotiana tabacum	Plant	**euk plant**	wt	353	1J93	0.256	2.3	apo	
Homo sapiens	Human	**euk animal**	F217Y	367	3GVW	0.274	2.8	apo	
Homo sapiens	Human	**euk animal**	Y164G	367	3GVV	0.262	2.8	apo	
Homo sapiens	Human	**euk animal**	Y164G	367	3GVR	0.246	2.2	apo	
Homo sapiens	Human	**euk animal**	D86G	367	1R3S	0.19	1.65	Coproporphyrin I	
Acinetobacter baumannii AB5075	Pseudomonadota	**prok g-**	wt	365	4ZR8	0.161	1.5	apo	
Homo sapiens	Human	**euk animal**	G156D	388	1JPK	0.238	2.2	apo	
Homo sapiens	Human	**euk animal**	F232L	388	1JPI	0.243	2.3	apo	
Homo sapiens	Human	**euk animal**	I260T	388	1JPH	0.235	2.1	apo	
Homo sapiens	Human	**euk animal**	K297N	367	3GW3	0.238	1.7	apo	
Homo sapiens	Human	**euk animal**	G318R	367	3GW0	0.24	2	apo	

Homo sapiens	Human	**euk animal**	wt	367	3GVQ	0.244	2.1	apo	
Pseudomonas aeruginosa	Pseudomonadota	**prok g-**	wt	363	4WSH	0.201	1.95	apo	
Homo sapiens	Human	**euk animal**	wt	367	1URO	0.233	1.8	apo	
Burkholderia thailandensis E264	Pseudomonadota	**prok g-**	wt	368	4EXQ	0.2	1.65	apo	
Homo sapiens	Human	**euk animal**	G168R	356	2Q6Z	0.222	2	apo	
Aquifex aeolicus	Aquificota	**prok g-**	wt	338	2EJA	0.27	1.9	apo	
Homo sapiens	Human	**euk animal**	G168R	356	2Q71	0.199	1.9	Coproporphyrin III	
Homo sapiens	Human	**euk animal**	wt	367	1R3Y	0.186	1.75	Coproporphyrin III	
Homo sapiens	Human	**euk animal**	Y164F	367	1R3W	0.189	1.7	Coproporphyrin III	
Homo sapiens	Human	**euk animal**	D86E	367	1R3V	0.187	1.9	Coproporphyrin I	
Homo sapiens	Human	**euk animal**	D86G	367	1R3T	0.2	1.7	Coproporphyrin III	
Homo sapiens	Human	**euk animal**	D86N	367	1R3R	0.192	1.85	apo	
Homo sapiens	Human	**euk animal**	wt	367	1R3Q	0.192	1.7	Coproporphyrin I	
Stenotrophomonas maltophilia	Pseudomonadota	**prok g-**	wt	365	6W2O	0.171	1.55	apo	

PpoX/HemY and CgoX/HemN (Coproporphyrinogen III oxidase)

Organism	Phylum	Taxonomy	Mutation?	Length	PDB code	R-free	Resolution [A°]	Ligand	Notes

Leishmania donovani	Euglenozoa	**euk**	wt	310	3EJO	0.267	2.3	apo	
Leishmania major	Euglenozoa	**euk**	wt	309	2QT8	0.193	1.75	apo	
Acinetobacter baumannii	Pseudomonadota	**prok g-**	wt	324	5EO6	0.168	1.45	apo	
Leishmania major	Euglenozoa	**euk**	wt	309	1VJU	0.18	1.4	apo	
Leishmania major	Euglenozoa	**euk**	wt	309	3DWS	0.259	2.5	Coproporphyrin III	
Leishmania major	Euglenozoa	**euk**	wt	309	3DWR	0.186	1.66	Coproporphyrin III	
Saccharomyces cerevisiae	Yeast	**euk**	wt	326	1TKL	0.258	2	apo	
Saccharomyces cerevisiae	Yeast	**euk**	wt	326	1TLB	0.282	2.4	apo	
Saccharomyces cerevisiae	Yeast	**euk**	wt	328	1TXN	0.245	1.7	apo	
Saccharomyces cerevisiae	Yeast	**euk**	wt	260	1TK1	0.249	1.9	apo	
Escherichia coli K-12	Pseudomonadota	**prok g-**	wt	457	1OLT	0.187	2.07	apo	
Homo sapiens	Human	**euk animal**	wt	346	2AEX	0.208	1.58	apo	
Leishmania naiffi	Euglenozoa	**euk**	wt	306	3E8J	0.245	2.27	apo	
Bacillus subtilis	Firmicutes	**prok g+**	wt	470	3I6D	0.293	2.9	FAD, Acifluorfen	Labelled as PpoX
Exiguobacterium sibiricum 255-15	Firmicutes	**prok g+**	wt	475	3LOV	0.241	2.06	apo	Labelled as PpoX

PpfC/HemH and CpfC/HemH (Coproporphyrin ferrochelatase)

**Organism**	**Phylum**	**Taxonomy**	**Mutation?**	**Length**	**PDB code**	**R-free**	**Resolution [A°]**	**Ligand**	**Notes**
Bacillus anthracis, str. Ames	Firmicutes	**prok g+**	wt	311	2C8J	0.281	2.1	apo	
Bacillus subtilis	Firmicutes	**prok g+**	Y13M	310	3GOQ	0.232	1.6	apo	
Bacillus subtilis	Firmicutes	**prok g+**	wt	309	3M4Z	0.199	1.94	Co^{2+}	
Bacillus subtilis	Firmicutes	**prok g+**	wt	310	1AK1	0.243	1.9	apo	
Bacillus subtilis	Firmicutes	**prok g+**	wt	306	1C9E	0.255	2.3	Cu^{2+} N-Methylmesoporphyrin complex	
Bacillus subtilis	Firmicutes	**prok g+**	wt	310	1LD3	0.274	2.6	Zn^{2+}	
Bacillus subtilis	Firmicutes	**prok g+**	wt	310	1N0I	0.273	2	Cd^{2+}	
Bacillus subtilis	Firmicutes	**prok g+**	wt	310	1C1H	0.231	1.9	N-Methylmesoporphyrin	
Bacillus subtilis	Firmicutes	**prok g+**	wt	310	2H1V	0.173	1.2	apo	
Bacillus subtilis	Firmicutes	**prok g+**	wt	309	1DOZ	0.216	1.8	apo	
Bacillus subtilis	Firmicutes	**prok g+**	wt	310	2HK6	0.22	1.71	Fe^{2+}	
Bacillus subtilis	Firmicutes	**prok g+**	wt	309	2AC2	0.257	2.5	Zn^{2+}	
Bacillus subtilis	Firmicutes	**prok g+**	H183C	309	2AC4	0.276	2.1	apo	

Bacillus subtilis	Firmicutes	**prok g+**	H183A	310	2H1W	0.259	2.6	apo	
Bacillus subtilis	Firmicutes	**prok g+**	wt	309	2Q2N	0.25	1.8	Deuteroporphyrin IX 2,4-disulfonic acid dihydrochloride	
Bacillus subtilis	Firmicutes	**prok g+**	H183C	309	2Q2O	0.219	2.1	Deuteroporphyrin IX 2,4-disulfonic acid dihydrochloride	
Bacillus subtilis	Firmicutes	**prok g+**	H183A	309	2Q3J	0.228	2.39	N-Methylmesoporphyrin	
Listeria monocytogenes	Firmicutes	**prok g+**	wt	312	6RWV	43622	0.201	1.64	apo
Listeria monocytogenes	Firmicutes	**prok g+**	wt	311	6SV3	43725	0.201	1.64	Coproheme
Listeria monocytogenes	Firmicutes	**prok g+**	R45L	311	8AW7	44802	0.213	2.64	Coproporphyrin III
Listeria monocytogenes	Firmicutes	**prok g+**	wt	311	8AT8	44923	0.182	1.51	Coproporphyrin III
Listeria monocytogenes	Firmicutes	**prok g+**	wt	311	8BBV	44848	0.242	2.19	Coproporphyrin III/2 min Fe2+ soak
Listeria monocytogenes	Firmicutes	**prok g+**	wt	311	8OMM	45016	0.233	2.15	Coproporphyrin III/3 min Fe2+ soak

Listeria monocytogenes	Firmicutes	**prok g+**	wt	311	8OFL	45021	0.229	2.1	Coproporphyrin III/4 min Fe2+ soak
Saccharomyces cerevisiae	Yeast	**euk**	wt	362	1LBQ	0.282	2.4	apo	
Saccharomyces cerevisiae	Yeast	**euk**	wt	362	1L8X	0.287	2.7	Co^{2+}	
Homo sapiens	Human	**euk**	E343K	359	2HRE	0.279	2.5	Protoporphyrin IX	
Homo sapiens	Human	**euk**	F337A	359	2PNJ	0.253	2.35	apo	
Homo sapiens	Human	**euk**	H263C	359	2PO5	0.24	2.2	apo	
Homo sapiens	Human	**euk**	H341C	359	2PO7	0.245	2.2	apo	
Saccharomyces cerevisiae	Yeast	**euk**	H235C	356	7L78	0.297	2.4	apo	
Homo sapiens	Human	**euk**	wt	359	1HRK	0.226	2	apo	
Homo sapiens	Human	**euk**	E343K	359	2QD1	0.261	2.2	Protoporphyrin IX	
Homo sapiens	Human	**euk**	F110A	359	2QD2	0.261	2.2	Fe-Protoporphyrin IX (Protoheme)	
Homo sapiens	Human	**euk**	M76H	370	4KMM	0.259	2.6	apo	
Homo sapiens	Human	**euk**	R115L	359	2HRC	0.242	1.7	apo	
Homo sapiens	Human	**euk**	wt	359	3HCN	0.236	1.6	Fe-Protoporphyrin IX (Protoheme), Hg^{2+}	
Homo sapiens	Human	**euk**	wt	359	3HCP	0.201	2	Fe-Deuteroporphyrin IX, Mn^{2+}	

Homo sapiens	Human	**euk**	wt	359	3HCR	0.25	2.2	Fe-Deuteroporphyrin IX, Ni^{2+}	
Homo sapiens	Human	**euk**	wt	359	3HCO	0.252	1.8	Fe-Protoporphyrin IX (Protoheme), Cd^{2+}	
Homo sapiens	Human	**euk**	wt	359	2QD3	0.242	2.2	Fe-Protoporphyrin IX (Protoheme)	
Homo sapiens	Human	**euk**	wt	359	2QD4	0.23	2	Mn^{2+}	
Homo sapiens	Human	**euk**	H240A	359	3AQI	0.214	1.7	apo	
Homo sapiens	Human	**euk**	F337R	359	4F4D	0.207	1.8	apo	
Homo sapiens	Human	**euk**	wt	359	2QD5	0.28	2.3	Protoporphyrin IX (Protoheme), Hg^{2+}	
Homo sapiens	Human	**euk**	E343D	359	4KLA	0.285	2.6	apo	
Homo sapiens	Human	**euk**	E343Q	359	4KLR	0.258	2.18	Fe-Protoporphyrin IX (Protoheme)	
Homo sapiens	Human	**euk**	E343D/F110A	370	4KLC	0.299	2.4	Fe-Protoporphyrin IX (Protoheme)	
Homo sapiens	Human	**euk**	S197C	359	4MK4	0.277	2.5	apo	

ChdC/HemQ (Coproheme decarboxylase)

**Organism**	**Phylum**	**Taxonomy**	**Mutation?**	**Length**	**PDB code**	**R-free**	**Resolution [A°]**	**Ligand**	**Notes**
Corynebacterium dipththeriae	Actinobacter	**prok g+**	Y135A	237	7Q4G	0.219	1.82	Coproheme	

Corynebacterium diptheriae	Actinobacter	**prok g+**	W183Y	237	7Q4F	0.182	2.15	Coproheme	
Listeria monocytogenes	Firmicutes	**prok g+**	wt	250	6FXQ	0.214	1.69	Coproheme, monovinyl monopropionate deuteroheme	
Listeria monocytogenes	Firmicutes	**prok g+**	wt	250	6FXJ	0.214	1.79	Coproheme	
Geobacillus stearothermophilus	Firmicutes	**prok g+**	wt	248	5T2K	0.176	1.8	Mn-Coproporphoryn III	
Listeria monocytogenes	Firmicutes	**prok g+**	wt	250	5LOQ	0.215	1.69	Coproheme	
Corynebacterium diptheriae	Actinobacter	**prok g+**	wt	237	6XUB	0.227	1.78	monovinyl monopropionate deuteroheme	
Corynebacterium diptheriae	Actinobacter	**prok g+**	wt	237	6XUC	0.223	1.87	Coproheme	
Geobacillus stearothermophilus	Firmicutes	**prok g+**	wt	248	1T0T	0.194	1.75	apo	
Geobacillus stearothermophilus	Firmicutes	**prok g+**	wt	248	6VSC		2.6	apo	Cryo EM
Geobacillus stearothermophilus	Firmicutes	**prok g+**	wt	248	6VSA		2.32	apo	Cryo EM
Listeria monocytogenes	Firmicutes	**prok g+**	wt	253	4WWS	0.229	2	apo	

Thermus thermophilus HB8	Deinococcota	**prok g-**	wt	249	1VDH	0.218	2	apo	

Frataxin

**Organism**	**Phylum**	**Taxonomy**	**Mutation?**	**Length**	**PDB code**	**R-free**	**Resolution [A°]**	**Ligand**	**Notes**
Chaetomium thermophilum	Fungus	**euk**	wt	126	6FCO	0.26	2.03	apo	FXN-like
Psychromonas ingrahamii	Pseudomonadota	**prok g-**	wt	105	4HS5	0.202	1.45	apo	
Escherichia coli	Pseudomonadota	**prok g-**	wt	106	1EW4	0.213	1.4	apo	
Saccharomyces cerevisiae	Yeast	**euk**	wt	123	2GA5			apo	NMR
Saccharomyces cerevisiae	Yeast	**euk**	wt	123	2FQL	0.305	3.01	apo	
Escherichia coli	Pseudomonadota	**prok g-**	wt	108	1SOY			apo	NMR, CyaY (Frataxin orthologue)
Saccharomyces cerevisiae	Yeast	**euk**	wt	123	4EC2	0.311	3	Fe^{2+}	
Saccharomyces cerevisiae	Yeast	**euk**	wt	123	3OEQ	0.292	2.96	apo	
Saccharomyces cerevisiae	Yeast	**euk**	wt	123	3OER	0.256	3.2	Co^{2+}	

Escherichia coli	Pseudomonadota	**prok g-**	wt	106	2P1X	0.222	1.42	Eu^{3+}	CyaY (Frataxin orthologue)
Escherichia coli	Pseudomonadota	**prok g-**	wt	106	2EFF	0.228	1.8	Co^{2+}	CyaY (Frataxin orthologue)
Psychromonas ingrahamii	Pseudomonadota	**prok g-**	wt	105	4LP1	0.26	1.8	Eu^{3+}	CyaY (Frataxin orthologue)
Psychromonas ingrahamii	Pseudomonadota	**prok g-**	wt	105	4LK8	0.211	1.49	Co^{2+}	CyaY (Frataxin orthologue)
Burkholderia cenocepacia	Pseudomonadota	**prok g-**	wt	112	4JPD	0.196	1.9	apo	CyaY (Frataxin orthologue)
Homo sapiens	Human	**euk animal**	wt	127	1EKG	0.22	1.8	apo	
Homo sapiens	Human	**euk animal**	wt	121	1LY7			apo	C term domain, NMR
Saccharomyces cerevisiae	Yeast	**euk**	wt	142	5TRE		15.6	apo	Cryo EM
Homo sapiens	Human	**euk animal**	R165C	129	3T3X	0.251	1.57	apo	Friedreich's ataxia variant
Homo sapiens	Human	**euk animal**	Q148G	129	3T3T	0.227	1.38	apo	
Homo sapiens	Human	**euk animal**	Q153A	129	3T3L	0.179	1.15	apo	
Homo sapiens	Human	**euk animal**	Q148R	129	3T3K	0.197	1.24	apo	Friedreich's ataxia variant
Homo sapiens	Human	**euk animal**	N146K	129	3T3J	0.245	1.7	apo	Friedreich's ataxia variant

Homo sapiens	Human	**euk animal**	W155F	129	3S5F	0.213	1.5	apo	
Homo sapiens	Human	**euk animal**	W155R	129	3S5E	0.197	1.31	apo	Friedreich's ataxia variant
Homo sapiens	Human	**euk animal**	W155A	129	3S5D	0.192	1.5	apo	
Homo sapiens	Human	**euk animal**	R165C	129	3T3X	0.251	1.57	apo	Friedreich's ataxia variant
Drosophila melanogaster	Fly	**euk animal**	wt	190	7N9I	0.18	1.4	apo	
Escherichia coli	Pseudomonadota	**prok g-**	wt	106	8HZ1	0.356	2	apo	CyaY (Frataxin orthologue)
Bacillus subtilis	Firmicutes	**prok g+**	wt	124	2OC6	0.217	1.75	apo	Hypothetical protein (NP_388456.1)
Lactobacillus casei	Firmicutes	prok g+	wt	123	2I8D	0.197	1.69	apo	Hypothetical protein (ZP_00384875.1)
Alkalihalobacillus halodurans	Firmicutes	**prok g+**	wt	118	2KL4			apo	NMR, Hypothetical protein, BLAST --> iron chaperon
